# A revised biopsychosocial model for explaining complexity to mental disorders

**DOI:** 10.1192/j.eurpsy.2025.251

**Published:** 2025-08-26

**Authors:** A. Fiorillo

**Affiliations:** University of Campania “Luigi Vanvitelli”, Naples, Italy

## Abstract

**Abstract:**

The biopsychosocial model was first conceptualized by George L. Engel in 1977 in order to explain the contribution of biological, social and psychological factors in determining mental (and physical) illnesses. This model gained new attention in recent years: while many authors consider it as a complete framework, others highlight its clinical, scientific and theoretic vagueness. A revised biopsychosocial model is being proposed, including causal interactions within and between biological, social and psychological factors as the benchmarks of the complexity of mental disorders.

**Disclosure of Interest:**

None Declared

